# Risk prediction models for chemotherapy-induced nausea and vomiting: a systematic review and meta-analysis

**DOI:** 10.3389/fonc.2026.1750558

**Published:** 2026-03-10

**Authors:** Xuemei Xie, Hang Li, Yue Li, Han Fu, Yunqiong Wang, Jia Cheng

**Affiliations:** 1 Sichuan Provincial Center for Mental Health, Sichuan Provincial People’s Hospital, School of Medicine, University of Electronic Science and Technology of China, Chengdu, China; 2 Department of Neonatology Nursing, West China Second Hospital of Sichuan University, Chengdu, China; 3 Department of Dermatology, Southwest Hospital, Army Medical University, Chongqing, China

**Keywords:** CINV, meta-analysis, prediction model, risk prediction, systematic review

## Abstract

**Objectives:**

To systematically review and critically appraise currently available risk prediction models for chemotherapy-induced nausea and vomiting (CINV).

**Methods:**

We searched nine electronic databases from inception to April 2025. Data extraction followed the CHARMS checklist. Risk of bias and applicability were assessed using the PROBAST tool, and reporting transparency was evaluated against the TRIPOD statement.

**Results:**

15 studies describing 16 distinct CINV risk prediction models were included. Reported area under the curve (AUC) values ranged from 0.629 to 0.850. Frequently incorporated predictors included age, gender, history of anticipatory nausea and vomiting, chemotherapy regimen, and number of chemotherapy cycles. All studies demonstrated a high risk of bias, primarily attributable to suboptimal data sources and inadequate reporting in the analytical domain. Meta-analysis of AUC values from eight development models yielded a pooled estimate of 0.74 (95% *CI*: 0.68-0.81), indicating moderate discrimination.

**Conclusions:**

Existing CINV risk prediction models exhibit significant methodological limitations and remain largely in the developmental phase. While common predictors emerge, controversies persist. Future research should prioritize developing novel models with larger sample sizes, rigorous methodology, multicenter external validation, enhanced clinical utility, and improved reporting transparency.

**Systematic Review Registration:**

https://www.crd.york.ac.uk/prospero/, identifier CRD42023395416.

## Introduction

As reported by the International Agency for Research on Cancer (IARC), close to 20 million new cancer cases were recorded in 2022, and population-based forecasts suggest that the annual incidence of new cancer cases is anticipated to rise to 35 million by 2050, representing a 77% increase compared to 2022 (1). This significant escalation not only highlights the considerable threat that cancer poses to human health but also reinforces its position as one of the foremost causes of mortality globally, posing a substantial challenge to public health efforts worldwide (2, 3). Among the range of cancer treatment strategies, chemotherapy has become one of the major modalities because of its notable effectiveness and widespread use, offering hope for survival to numerous individuals battling cancer (4). Nevertheless, despite the therapeutic benefits of chemotherapy, it often leads to various adverse effects. One of the most prevalent reactions is chemotherapy-induced nausea and vomiting (CINV), which is frequently cited as one of the most distressing and alarming symptoms experienced by patients (5, 6). Research indicates that the likelihood of experiencing CINV during chemotherapy can reach as high as 60% to 80% (7–10). Severe episodes of nausea and vomiting may lead to malnutrition, dehydration, and disturbances in water and electrolyte balance, adversely affecting patients’ overall quality of life (11, 12). Additionally, some patients might experience anxiety, depression, and reduced compliance with treatment, which not only detracts from therapeutic efficacy but also increases the financial strain on patients, sometimes even leading them to abandon treatment altogether (13, 14).

At present, the main strategy in clinical settings for managing this symptom involves the administration of antiemetic medications as an intervention. Despite notable progress in the development of antiemetic drugs aimed at preventing and treating CINV, around 30% of cancer patients continue to experience inadequate relief from nausea and vomiting symptoms (15). In particular, controlling nausea symptoms, especially those related to delayed CINV (which occurs 24 to 120 hours post-chemotherapy), is still insufficient (16). This limitation arises because the current choice of preventive medication regimens for CINV largely depends on the emetic potential of the chemotherapy used, failing to take individual patient factors into account, which complicates the personalized management of CINV (17). The onset of CINV is influenced by various factors, and forecasting the likelihood of nausea and vomiting symptoms solely based on the emetic potential of chemotherapy lacks both scientific foundation and dependability, potentially resulting in the misuse of medications (17). Clinical guidelines advocate for a preventive strategy to handle CINV symptoms, employing a structured, time-sensitive, and combinatory approach for tailored treatment (18). Consequently, enabling timely and accurate predictions regarding CINV can greatly assist healthcare providers in implementing preventive measures, facilitating the formulation of precise and personalized antiemetic strategies for patients undergoing chemotherapy (38). Ultimately, this approach enhances the quality of life for patients, leads to improved treatment outcomes, and enriches their overall medical experience.

CINV risk prediction models integrate diverse predictors to estimate the likelihood of CINV occurrence, presenting complex data in a clinically interpretable format (19). These tools facilitate the identification of high-risk patients, providing clinicians with valuable insights for developing individualized management plans (20). While numerous CINV prediction models exist, reported predictive performance varies considerably, and a systematic assessment of their methodological quality, clinical applicability, and reporting transparency has been lacking. This systematic review therefore aims to identify and critically appraise existing CINV risk prediction models for cancer patients, offering a foundation for clinical implementation and future model development and validation.

## Methods

This review adhered to the Preferred Reporting Items for Systematic Reviews and Meta-Analyses (PRISMA) guidelines (21) and is registered on PROSPERO (CRD42023395416). As a review incorporating meta-analysis, ethical committee approval was not required.

### Search strategy

Searches were conducted in SinoMed, PubMed, Web of Science, The Cochrane Library, CINAHL, Embase, CNKI, Wanfang Database, and VIP from inception to April 20, 2025. Key search terms included: “chemotherapy induced nausea vomiting”, “chemotherapy nausea”, “chemotherapy vomiting”, “chemotherapy emesis”, “neoplasms”, “tumor”, “cancer”, “risk prediction model”, “risk factor”, “predictor”, “model”, “risk Score”. Detailed search strategies are provided in **Supplementary Appendix A**. Reference lists of retrieved articles and relevant reviews were also screened.

### Inclusion and exclusion criteria

#### Inclusion criteria

(1) Study population: Cancer patients receiving chemotherapy. (2) Study focus: Development and/or validation of CINV risk prediction models (requiring ≥ 2 predictors). (3) Study design: Cohort, cross-sectional, or case-control studies.

#### Exclusion criteria

(1) Studies solely identifying risk factors without developing/validating a prediction model. (2) Articles not published in English or Chinese. (3) Conference abstracts, letters, news reports, reviews, editorials, or non-original research. (4) Unavailable full text.

### Study selection

Two investigators (XMX and HF) independently screened retrieved records using Endnote X9. Duplicates were removed first. Titles and abstracts were then assessed for eligibility based on inclusion/exclusion criteria. Potentially eligible full texts were reviewed, and reference lists were scanned for additional studies. Disagreements were resolved through discussion between the two reviewers or consultation with a third author (JC).

### Data extraction

A standardized data extraction form, adapted from the CHARMS checklist, was used independently by two reviewers. Extracted information included: (1) Basic information: Author, publication year, study design, participants, data source, sample size. (2) Model information: Variable selection method, model development technique, validation type, performance metrics (e.g., AUC), missing data handling, continuous variable processing, final model predictors, model presentation format. Cross-checking ensured accuracy and consistency. The extraction template is detailed in **Supplementary Appendix B**.

### Quality assessment

To evaluate the quality and potential bias risk of the included studies, two assessment tools were employed: the Grading of Recommendations Assessment, Development, and Evaluation (GRADE) (22) and the available version of the Prediction model Risk Of Bias Assessment Tool (PROBAST) (23). (**Supplementary Appendix C**). Transparent Reporting of a Multivariate Prediction Model for Individual Prognosis or Diagnosis (TRIPOD) (24) for reporting transparency (**Supplementary Appendix D**).

### Data analysis

Meta-analysis of the area under the curve (AUC) for validated models was performed using Stata 17.0. Heterogeneity was quantified using the *I*^2^ statistic and the Cochrane Q test (*I*² values: 25% low, 50% moderate, 75% high heterogeneity) (25). Fixed-effects or random-effects models were applied based on the level of heterogeneity observed. Publication bias was assessed using Egger’s test, where a *p*-value > 0.05 suggests a low likelihood of bias (26).

## Result

### Study selection

**Figure 1:**
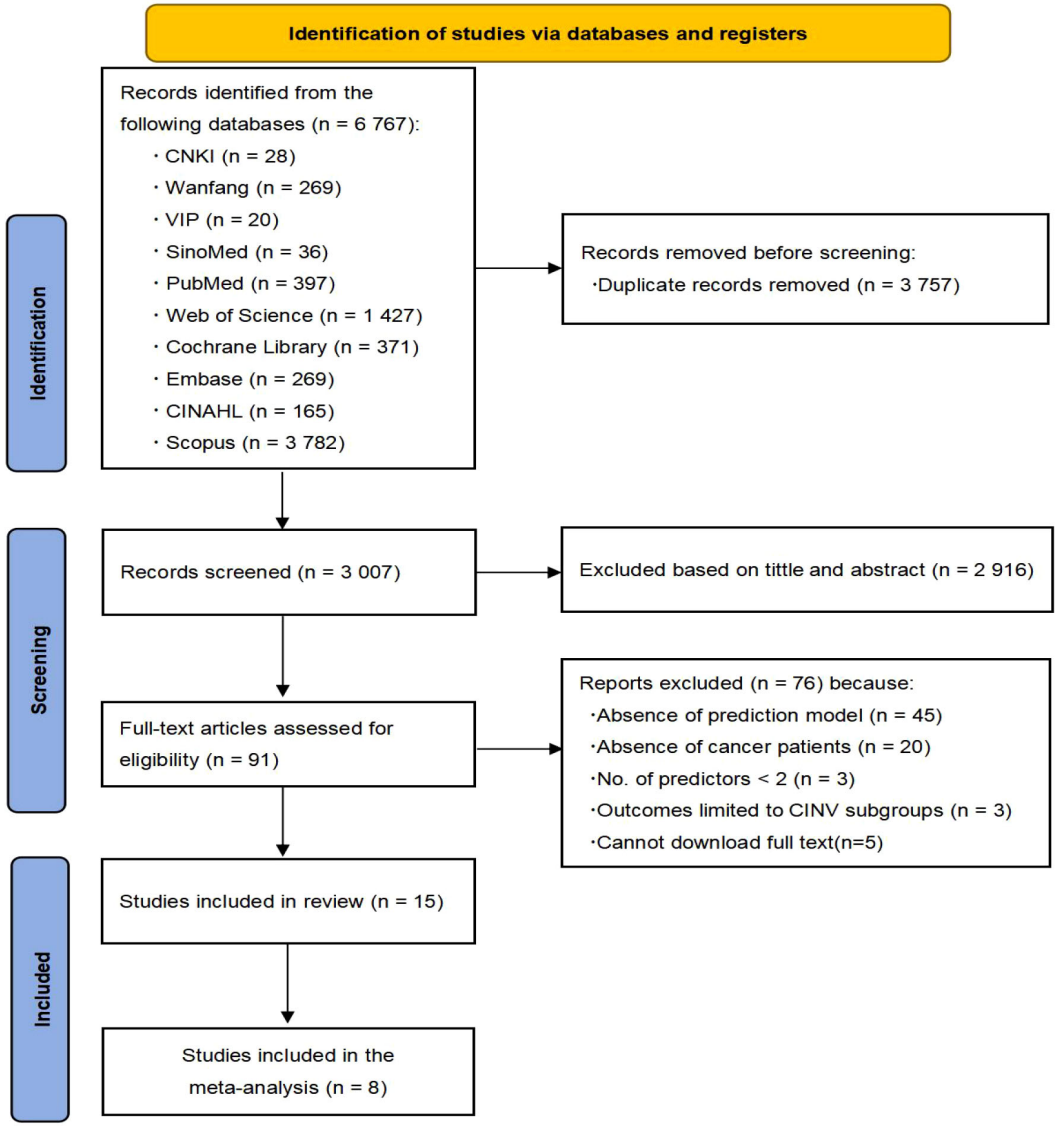
Flowchart of study inclusion.

6767 literature were obtained through database search. After the initial screening of removing duplicates, reading titles and abstracts, 6673 articles were excluded. 91 studies were underwent full-text review. 45 studies were excluded for lacking a prediction model (risk factor studies only), 20 studies for inconsistent populations, three studies had less than two predictors, three studies had outcomes limited to subgroups, and five studies could not download the full text. 15 studies describing 16 models were ultimately included. The PRISMA flowchart (**Figure 1**) details the selection process.

### Study characteristics

The 15 included studies (20, 27–40) comprised 12 model development studies, 2 validation studies, and 1 model optimization study. Nine studies originated from China (5 published in Chinese), three from Canada, and one each from the USA, UK, and South Korea. 11 studies employed prospective designs, four were retrospective. In terms of participants, one study was only in patients undergoing chemotherapy for gastric cancer, two studies were only in patients undergoing chemotherapy for breast cancer, and the remaining 12 studies were in patients undergoing chemotherapy for cancer. 14 studies predicted CINV occurrence; one predicted broader chemotherapy-induced adverse drug reactions (ADRs). Sample sizes ranged from 97 to 6124. The detailed basic characteristics of the included studies are shown in **Table 1**.

**Table 1:** Overview of basic data of the included studies.

Author (Year)	Country	Study design	Participants	Data source	Follow-up time	Main outcome	Model type	CINV cases (%)	Sample size
Lu xiangyuan* (2023) (34)	China	Prospective cohort study	Breast cancer patients	Fujian provincial cancer hospital	Day 1 to day 7 after chemotherapy	CINV	Model development	134(39.5%)	339
Huang guiling* (2022) (32)	China	Prospective cohort study	Gastric cancer patients	Fujian provincial cancer hospital	Day 1 to day 5 after chemotherapy	CINV	Model development	71(37.6%)	189
Zhang yuqing* (2023) (40)	China	Prospective cohort study	Cancer patients	Second affiliated hospital of Xuzhou medical university	-	CINV	Model development	134(41.9%)	320
Deng B (2022) (20)	China	Prospective cohort study	Cancer patients	Chongqing 12 third class A hospitals	Day 1 to day 5 after chemotherapy	CINV	Model development	639(28.8%)	2215
Cao zongping* (2021) (28)	China	Prospective cohort study	Cancer patients	Second xiangya hospital central south university	Information during hospitalization and for 5 consecutive days after the end of chemotherapy.	CINV	Model development	A:131(43.7%) B:207(69.0%)	300
Hu zhihuang (2016) (31)	China	Prospective cohort study	Cancer patients	multi-institutional investigations in Asian countries	1 Week	CINV	Model development and validation	C:204 (37.2%) D:80 (25.5%)	C:548 D:314
Bouganim (2012) (27)	Canada	Prospective cohort study	Cancer patients	Ottawa hospital cancer center	By 24-hour and 5-day telephone callbacks after chemotherapy in every cycle.	CINV	Model validation	A:17(17.3%) B:18(18.4%)	98
Mosa et al. (2020a) (36)	USA	Retrospective study	Cancer patients	University of Missouri ellis fischel cancer center	-	CINV	Model development	A:1519(49.75%) B:1552(50.54%)	6124
Dranitsaris G (2017) (30)	Canada	Retrospective study	Cancer patients	Outpatient service	The first 24 h and from days 2 to 5 following chemotherapy	CINV	Model development	1771(42.2%)	4197
Molassiotis (2013) (35)	UK	Prospective cohort study	Cancer patients	16 cancer centers in the UK	1 week before chemotherapy to 3 weeks before chemotherapy	CINV	Model development and validation	148(44%)	336
Huang Xinjuan (2021) (33)	China	Prospective cohort study	Breast cancer patients	Hunan cancer hospital, Hunan provincial maternal and child health care hospital	Days 2 and 6 after chemotherapy.	CINV	Model development and validation	C:137(41%) D:30(45%)	C:334 D:66
Zhang Linlin (2023) (39)	China	Prospective cohort study	Cancer patients	Tianjin medical university general hospital, Department Tianjin medical university cancer institute	Day 1 to day 15 after chemotherapy	CINV	Model development and validation	563(42%)	1356
Dranitsaris G (2013) (29)	Canada	Prospective cohort study	Cancer patients	Ottawa hospital cancer center, Irving Greenburg family cancer center	Day 1 to day 5 after chemotherapy	CINV	Model validation	A:13(13.5%) B:21(21.6%)	97
On J (2022) (37)	Korea	Retrospective study	Cancer patients	Tertiary teaching hospital	-	Chemotherapy-induced ADRs	Model development	685(73.3%)	935
Zhang jingyue (2023) (38)	China	Retrospective study	Cancer patients	Tianjin medical university general hospital	Day 1 to day 14 after chemotherapy	CINV	Model development	227(30%)	756

A = acute CINV (0-24h); B = delayed CINV (25-120h); C = model development; D = model validation; ADRs = a diverse drug reactions.

*Study was published in Chinese.

### Model information

The 16 identified models comprised two for acute CINV (0-24h), two for delayed CINV (25-120h), and twelve for non-specific CINV. Development methods included logistic regression (7 studies), machine learning (4 studies), and multiple generalized estimation equations (2 studies). Age (13 models) and history of CINV (11 models) were the most common predictors. Reported AUC or C-statistic values spanned 0.629 to 0.850. Calibration was reported for ten models, primarily using the Hosmer-Lemeshow test. Final models incorporated 4 to 19 variables and were presented as formulas, nomograms, risk scoring systems, or applications. Detailed information is shown in **Supplementary Appendix E**.

### Model validation

Among the included studies, most models underwent internal or external validation. Among them, only three studies performed external validation, eight studies performed internal validation, while the models by Huang et al. (33) and Hu et al. (31) included both internal and external validation. The model developed by Deng and Zhang (20, 40) did not undergo any validation after its development.

### Quality evaluation

#### Risk of bias assessment (PROBAST)

All studies were rated as having a high overall risk of bias, indicating methodological deficiencies in development or validation. The PROBAST results of the included studies are detailed in **Table 2**.

**Table 2:** PROBAST results of the included studies.

Author (Year)	Study type	ROB				Applicability			Overall	
		Participants	Predictors	Outcome	Analysis	Participants	Predictors	Outcome	ROB	Applicability
Lu xiangyuan* (2023) (34)	A	−	+	+	−	−	+	+	−	−
Huang guiling* (2022) (32)	A	−	+	+	−	−	+	+	−	−
Zhang yuqing* (2023) (40)	A	+	+	−	−	+	+	+	−	+
Deng benmin* (2022) (20)	A	+	+	−	−	+	+	+	−	+
Cao zongping* (2021) (28)	A	+	?	−	−	−	+	+	−	−
Hu zhihuang (2016) (31)	B	+	+	−	−	+	+	+	−	+
Bouganim (2012) (27)	C	+	+	−	−	+	+	+	−	+
Mosa et al. (2020a) (36)	A	−	?	−	−	+	+	+	−	+
Dranitsaris G (2017) (30)	A	−	+	−	−	+	+	+	−	+
Molassiotis (2013) (35)	B	+	+	−	−	+	+	+	−	+
Huang Xinjuan (2021) (33)	B	−	+	−	−	+	+	+	−	+
Zhang Linlin (2023) (39)	B	+	+	−	−	+	+	+	−	+
Dranitsaris G (2013) (29)	C	+	+	−	−	+	+	+	−	+
Jeongah On (2021)	A	−	+	−	−	+	+	+	−	+
Zhang jingyue (2023) (38)	A	−	+	−	−	+	+	+	−	+

PROBAST, prediction model risk of bias assessment tool; ROB, risk of bias.

A indicates “development only”; B indicates “development and validation in the same publication”; C indicates “ validation only”;

“+” indicates low ROB/low concern regarding applicability; “-” indicates high ROB/high concern regarding application; “?” indicates unclear ROB/unclear concern regarding applicability.

* Study was published in Chinese.

In the participant domain, a total of seven studies were categorized as high risk, while four studies were assessed as potentially biased due to possible recall bias or misclassification of outcome events in retrospective research. Additionally, certain crucial predictors related to the development of CINV in patients receiving cancer treatment may not have been adequately documented in medical records or may have been influenced by the inconsistency in training among assessors (30, 36–38). Three studies focused narrowly on specific cancers. Two were only for breast cancer chemotherapy patients (33, 34) and one was only for gastric cancer chemotherapy patients (32). Within the predictor domain, bias risk was unclear for two studies due to uncertain blinding during predictor assessment (28, 36). In the outcome domain, Three studies were high risk for not specifying the interval between predictor assessment and outcome evaluation (36, 37, 40). Eleven studies included previous history of nausea and vomiting as a predictor, rated high risk as this may inflate predictor-outcome associations and model performance estimates. One study lacked blinding between predictor and outcome assessment (39).

In the analysis domain, all studies demonstrated high risk. Key issues included: (1) Seven development studies had insufficient sample sizes (<20 events per variable (EPV)); two validation studies had <100 cases. (2) Seven studies categorized continuous variables without justification. (3) Nine studies failed to report missing data; six excluded missing cases entirely, risking bias. (4) Eight studies used univariate screening for variable selection. (5) Five studies omitted reporting model calibration or discrimination assessment. (6) Two studies did not report or consider the risk of bias due to overfitting, underfitting and best-fitting of the prediction model. (7) Two studies failed to report on internal validation.

In the model applicability domain, three studies were classified as high risk and the remaining studies as low risk. Because three studies were considered to be at high risk of applicability in the study area, two studies included participants limited to breast cancer and one study included participants limited to gastric cancer.

#### Transparent reporting assessment (TRIPOD)

**Figure 2:**
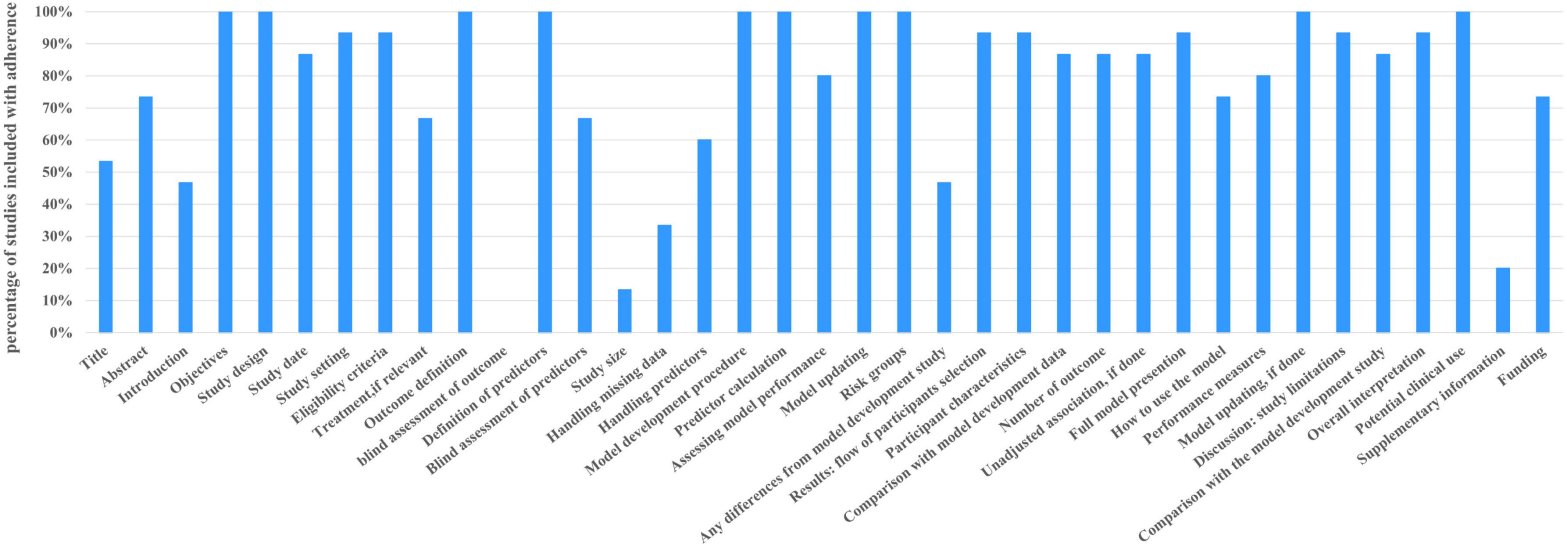
Included studies were assessed according to the TRIPOD.

In terms of transparent reporting assessment, a systematic evaluation of the included 15 studies was conducted based on the TRIPOD statement (**Figure 2**). The results showed that the total TRIPOD scores of these studies ranged from 21 to 31 (out of a maximum of 37 points). Among all 37 reporting items, six items had a transparent reporting completeness rate below 50%. From low to high, they were blind assessment of outcome, study size, supplementary information, handling missing data, any differences from model development study, and introduction. Objectives, study design, outcome definition, definition of predictors, model development procedure, predictor calculation, and potential clinical use had 100% transparency reporting completeness rate. Detailed information is shown in **Supplementary Appendix F**.

### Meta-analysis of development models included in the review

**Figure 3:**
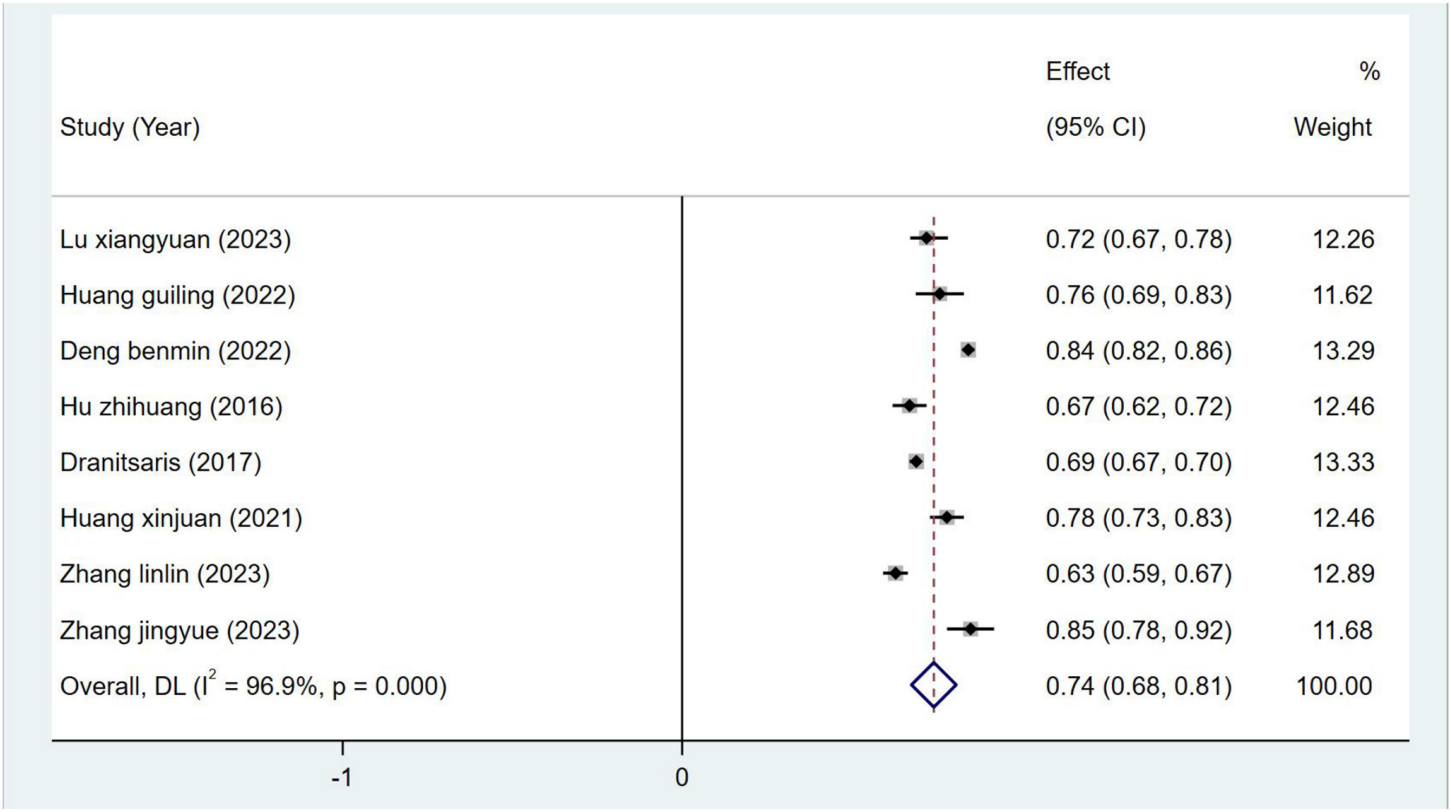
Forest plot of the random effects meta-analysis of pooled AUC estimates for 8 development models.

Due to insufficient reporting on development details in some studies, only eight studies were suitable for meta-analysis. The pooled AUC, calculated using a random-effects model, was 0.74 (95% *CI*: 0.68-0.81) (**Figure 3**). The *I*^2^ value was 96.9% (*P* < 0.001), indicating a high degree of heterogeneity among the studies. Begg’s test (*P* = 0.621 > 0.05), and Egger’s test (*P* = 0.930 > 0.05) suggesting no statistically significant publication bias. Detailed information can be found in **Supplementary Appendix G**.

### Subgroup analysis to explore heterogeneity

To explore the potential sources of high heterogeneity observed in the meta-analysis, we performed subgroup analyses based on chemotherapy regimen emetogenicity, cancer type, model development method, and CINV definition and assessment tool. The analysis revealed that heterogeneity was markedly reduced or absent in more homogeneous subgroups. Specifically, studies focusing solely on patients receiving moderately emetogenic chemotherapy (*I*²= 0.0%, *P* = 0.379) or highly emetogenic chemotherapy (*I*²= 0.0%, *P* = 0.788) demonstrated negligible heterogeneity, suggesting that variations in chemotherapy emetogenicity are a major contributor to the overall heterogeneity. Similarly, models developed using the GEE method and the models only for breast cancer patients showed low heterogeneity. In contrast, high heterogeneity persisted in subgroups encompassing mixed cancer types, diverse chemotherapy regimens, or studies using multivariable logistic regression or the CINV assessment tool. Detailed information is shown in **Supplementary Appendix H**.

## Discussion

As a principal method for tumor treatment, chemotherapy typically offers beneficial outcomes, albeit with a range of adverse effects. One of the most significant side effects for patients is CINV (41). Accurate prediction and effective prevention of CINV are therefore crucial, not only for improving patients’ quality of life and overall treatment outcomes but also for guiding rational clinical drug use and optimizing healthcare resource allocation (42). This systematic review comprehensively evaluates the methodological quality and predictive performance of existing CINV risk prediction models, aiming to provide direction for future research.

This review included 15 studies involving 16 CINV risk prediction models. These models demonstrated fair to good discriminatory ability during their development or validation phases, with reported AUC values ranging from 0.629 to 0.843. However, assessment using the PROBAST indicated that all included studies were judged to be at a high risk of bias. The bias primarily stemmed from the analysis domain, manifesting in issues such as insufficient sample size, suboptimal methods for variable selection, and inadequate handling of missing data and continuous variables (20, 27, 28). Concurrently, transparent reporting assessment to the TRIPOD guidelines revealed shortcomings in reporting key methodological details across all studies, which further increases the uncertainty of the model results and limits their generalizability (24).

To further quantify the overall performance of the models, we conducted a meta-analysis of the AUC from 8 development models. The results showed a pooled AUC of 0.74 (95% CI: 0.68-0.81). This value suggests that the existing models possess moderate predictive performance overall, yet its interpretation demands considerable caution. The reasons are twofold. Firstly, the meta-analysis revealed substantial heterogeneity. Further subgroup analysis indicated that this heterogeneity mainly originated from the diversity in clinical contexts and methodologies across studies, particularly differences in the emetogenicity levels of chemotherapy regimens (20, 38). In subgroups containing only highly or moderately emetogenic chemotherapy regimens, the heterogeneity became negligible. This strongly indicates that the predictive performance of the models is highly dependent on the specific treatment context (34). Consequently, the pooled AUC value should be regarded more as a reference reflecting an “average” or “optimistic upper limit” of the models’ discriminatory ability, rather than a reliable performance metric universally applicable to all clinical scenarios (43). Secondly, the widespread high risk of bias seriously undermines the credibility of this pooled estimate. Biasing factors such as the risk of overfitting due to small sample sizes (28, 32, 34), the simple exclusion of cases with missing data (31, 32, 40), the loss of information caused by inappropriate categorization of continuous predictors (30, 33, 39), and the prevalent reliance on univariate statistical significance for variable selection (20, 28, 34, 36) can all lead to an overestimation of model performance on development data and significantly limit their applicability to new populations. Therefore, considering both the high heterogeneity and high risk of bias, there is currently no sufficiently robust CINV prediction tool ready for direct and widespread clinical application (37). Existing models are better viewed as promising yet methodologically immature prototypes, whose efficacy and interpretation are highly dependent on the specific development context (44).

Despite these limitations, insights from the PROBAST assessment indicate that current studies still offer valuable experience and clear directions for improvement in developing more reliable prediction models. On the methodological front, future research must strictly adhere to best practices in prediction model studies. Ensuring an adequate sample size and a sufficient events-per-variable ratio is fundamental to preventing overfitting and enhancing model stability (39, 45, 46). For variable handling, robust methods such as multiple imputation should be employed for managing missing data, and arbitrary dichotomization of continuous predictors should be avoided (32, 47, 48), as such categorization leads to information loss and diminished predictive power (36, 49, 50). Variable selection strategies should move beyond simple univariate screening, with greater emphasis placed on the assessment and reporting of model calibration (20, 49, 51). Most critically, models must undergo rigorous external validation, preferably in populations that differ from the development cohort, to genuinely assess their generalizability and clinical transportability (39, 43, 52). Regarding the choice of modeling techniques, while logistic regression remains predominant, machine learning techniques have been explored by researchers such as Cao et al., Mosa et al., Zhang et al., and Jeongah et al. Machine learning methods often outperform traditional logistic regression in capturing complex patterns (53, 54) and may address challenges encountered in some studies, such as sample size constraints and complex interactions among predictors (28). However, the prevalent “black-box” nature of current machine learning models poses significant interpretability challenges. Consequently, the design, deployment, and iterative integration of explainable artificial intelligence principles should be emphasized within the development workflow (55). In summary, the choice of methodology should ultimately be driven by the specific clinical question, data characteristics, and practical requirements for model interpretability.

Furthermore, to enhance the clinical translational value of the models, future variable selection can be deeply integrated with evidence from real-world clinical studies (56). This review highlights common predictive factors such as age, sex, and prior history of CINV, which align with clinical experience and existing guidelines (40, 57–59). However, recent observational studies focusing on vulnerable populations have revealed more nuanced and potentially modifiable predictors. For example, a retrospective analysis by Yao et al. in elderly gynecological cancer patients not only confirmed the role of treatment-related factors like chemotherapy regimen and antiemetic prophylaxis but also identified pre-chemotherapy anxiety levels and sleep duration as modifiable independent predictors (56). This underscores the necessity of incorporating psychosocial factors and lifestyle indicators, especially in elderly populations. Additionally, the inclusion of body mass index (BMI) as a predictor by Mosa et al. (36) is consistent with findings from real-world study like Kawazoe et al. (60). Emerging evidence on genetic factors such as AB-CB1 gene, dopamine D2 receptor gene, serum 5-hydroxytryptamine 3C receptor gene, and catechol-o-methyltransferase gene polymorphisms (61) and laboratory markers such as serum sodium levels (62) also provides new directions for improving model precision. Future model development should prioritize the integration of these patient-specific variables supported by real-world evidence (39, 56). Leveraging advanced modeling techniques like machine learning to handle nonlinear interactions among multi-dimensional predictors can help bridge the gap between algorithm-driven model development and clinically evidence-based risk factor research, thereby enhancing the models’ accuracy, clinical relevance, and practical utility (63, 64).

It is important to note that the risk prediction models included in this study cover acute, delayed, and non-specific models. Although all are used for predicting CINV risk, each has its own strengths, weaknesses, and applicable scenarios. Acute CINV models allow risk assessment before chemotherapy, facilitating the formulation of individualized prevention strategies (28), However, their predictive accuracy can be limited by individual variability and data constraints (29). Delayed CINV models focus on the high-risk period 2-5 days after chemotherapy, addressing the prediction gap in the post-treatment phase (27), yet they similarly face challenges regarding prediction accuracy and data availability (28). Non-specific CINV risk prediction models integrate risks for both acute and delayed events, better aligning with the need for comprehensive clinical management (30), though their complexity may limit bedside application. In clinical practice, model selection requires comprehensive consideration of their features and individual patient circumstances. It is essential to emphasize that these models serve only as reference tools for healthcare providers and patients, not as sole bases for decision-making.

## Limitations

There are certain limitations to this review. Firstly, the inclusion of studies published only in English and Chinese may introduce language bias. Secondly, the heterogeneity and limited number of development models included in the meta-analysis represent another limitation. Finally, methodological shortcomings and incomplete reporting transparency in the original studies, as identified by the PROBAST and TRIPOD assessments, further constrained the depth of our analysis and may introduce additional biases.

## Conclusion

This systematic review evaluated 15 studies encompassing 16 CINV risk prediction models. Existing CINV risk prediction models demonstrate moderate predictive performance. However, their clinical application value is limited by high heterogeneity, a pervasive high risk of bias, and insufficient integration of predictors with real-world evidence. Future research needs to focus on methodological optimization, including ensuring adequate sample size, standardizing variable handling and missing data management, strengthening external validation, integrating clinically validated predictors from real-world settings especially modifiable factors in vulnerable populations and balancing model predictive performance with clinical interpretability. These efforts are essential for developing more reliable and practical individualized CINV risk prediction tools.

## Data Availability

The original contributions presented in the study are included in the article/**Supplementary Material**. Further inquiries can be directed to the corresponding author.

## References

[1] Bray F Laversanne M Sung H Ferlay J Siegel RL Soerjomataram I . Global cancer statistics 2022: GLOBOCAN estimates of incidence and mortality worldwide for 36 cancers in 185 countries. CA Cancer J Clin. (2024) 74:229–63. doi: 10.3322/caac.21834, PMID: 38572751

[2] Franzoi MA Di Meglio A Michiels S Gillanders E Gaudin C Martin AL . Patient-reported quality of life 6 years after breast cancer. JAMA Netw Open. (2024) 7:e240688. doi: 10.1001/jamanetworkopen.2024.0688, PMID: 38421653 PMC10905303

[3] Naghavi M Abajobir A Abbafati C . Global, regional, and national age-sex specific mortality for 264 causes of death 1980-2016: a systematic analysis for the Global Burden of Disease Study 2016. Lancet. (2017) 390:1151–210. doi: 10.1016/S0140-6736(17)32152-9, PMID: 28919116 PMC5605883

[4] Stine ZE Schug ZT Salvino JM Dang CV . Targeting cancer metabolism in the era of precision oncology. Nat Rev Drug Discov. (2022) 21:141–62. doi: 10.1038/s41573-021-00339-6, PMID: 34862480 PMC8641543

[5] Heckroth M Luckett RT Moser C Parajuli D Abell TL . Nausea and vomiting in 2021: A comprehensive update. J Clin Gastroenterol. (2021) 55:279–99. doi: 10.1097/MCG.0000000000001485, PMID: 33471485 PMC7933092

[6] Hernandez Torres C Mazzarello S Ng T Dranitsaris G Hutton B Smith S . Defining optimal control of chemotherapy-induced nausea and vomiting-based on patients’ experience. Support Care Cancer. (2015) 23:3341–59. doi: 10.1007/s00520-015-2801-y, PMID: 26108169

[7] Al Qadire M . Chemotherapy-induced nausea and vomiting: Incidence and management in Jordan. Clin Nurs Res. (2018) 27:730–42. doi: 10.1177/1054773817704586, PMID: 28388860

[8] Hesketh PJ . Chemotherapy-induced nausea and vomiting. N Engl J Med. (2008) 358:2482–94. doi: 10.1056/NEJMra0706547, PMID: 18525044

[9] Sun Y Zheng Y Yang X Xie K Du C He L . Incidence of chemotherapy-induced nausea and vomiting among cancer patients receiving moderately to highly emetogenic chemotherapy in cancer centers in Sichuan, China. J Cancer Res Clin Oncol. (2021) 147:2701–8. doi: 10.1007/s00432-021-03554-1, PMID: 33586045 PMC11802099

[10] Vazin A Eslami D Sahebi E . Evaluating the antiemetic administration consistency to prevent chemotherapy-induced nausea and vomiting with the standard guidelines: a prospective observational study. Ther Clin Risk Manag. (2017) 13:1151–7. doi: 10.2147/TCRM.S133820, PMID: 28919769 PMC5592905

[11] Neymark N Crott R . Impact of emesis on clinical and economic outcomes of cancer therapy with highly emetogenic chemotherapy regimens: a retrospective analysis of three clinical trials. Support Care Cancer. (2005) 13:812–8. doi: 10.1007/s00520-005-0803-x, PMID: 15834590

[12] Sommariva S Pongiglione B Tarricone R . Impact of chemotherapy-induced nausea and vomiting on health-related quality of life and resource utilization: A systematic review. Crit Rev Oncol Hematol. (2016) 99:13–36. doi: 10.1016/j.critrevonc.2015.12.001, PMID: 26697988

[13] Encinosa W Davidoff AJ . Changes in antiemetic overuse in response to choosing wisely recommendations. JAMA Oncol. (2017) 3:320–6. doi: 10.1001/jamaoncol.2016.2530, PMID: 27632203

[14] Sun CC Bodurka DC Weaver CB Rasu R Wolf JK Bevers MW . Rankings and symptom assessments of side effects from chemotherapy: insights from experienced patients with ovarian cancer. Support Care Cancer. (2005) 13:219–27. doi: 10.1007/s00520-004-0710-6, PMID: 15538640

[15] Hsieh RK Chan A Kim HK Yu S Kim JG Lee MA . Baseline patient characteristics, incidence of CINV, and physician perception of CINV incidence following moderately and highly emetogenic chemotherapy in Asia Pacific countries. Support Care Cancer. (2015) 23:263–72. doi: 10.1007/s00520-014-2373-2, PMID: 25120009

[16] Zong X Zhang J Ji X Gao J Ji J . Patterns of antiemetic prophylaxis for chemotherapy-induced nausea and vomiting in China. Chin J Cancer Res. (2016) 28:168–79. doi: 10.21147/j.issn.1000-9604.2016.02.04, PMID: 27199514 PMC4865609

[17] Rha SY Song SK Lee CE Park Y Lee J . Gaps exist between patients’ experience and clinicians’ awareness of symptoms after chemotherapy: CINV and accompanying symptoms. Support Care Cancer. (2016) 24:4559–66. doi: 10.1007/s00520-016-3295-y, PMID: 27278273

[18] Chen X Chen Y Qin S . Treatment of chemotherapy-induced nausea and vomiting based on guideline. Clin Medication J. (2014) 12:7–11. doi: 10.3969/j.issn.1672-3384.2014.05.002, PMID: 35900448

[19] Zheng L Zhang R . Research progress on fit evaluation methods of disease risk prediction models. Chin Health Stat. (2015) 32:544–6.

[20] Den B Chen Y Bian Z Ju J Zhou Y Zhang H . Construction of a risk prediction model for chemotherapy-related nausea and vomiting. Chin Nurs Manage. (2022) 22:1384–90. doi: 10.3969/j.issn.1672-1756.2022.09.022, PMID: 35900448

[21] Liberati A Altman DG Tetzlaff J Mulrow C Gøtzsche PC Ioannidis JP . The PRISMA statement for reporting systematic reviews and meta-analyses of studies that evaluate health care interventions: explanation and elaboration. PloS Med. (2009) 6:e1000100. doi: 10.1371/journal.pmed.1000100, PMID: 19621070 PMC2707010

[22] Guyatt GH Oxman AD Vist GE Kunz R Falck-Ytter Y Alonso-Coello P . GRADE: an emerging consensus on rating quality of evidence and strength of recommendations. BMJ. (2008) 336:924–6. doi: 10.1136/bmj.39489.470347.AD, PMID: 18436948 PMC2335261

[23] Wolff RF Moons KGM Riley RD Whiting PF Westwood M Collins GS . PROBAST: A tool to assess the risk of bias and applicability of prediction model studies. Ann Intern Med. (2019) 170:51–8. doi: 10.7326/M18-1376, PMID: 30596875

[24] Collins GS Reitsma JB Altman DG Moons KG . Transparent reporting of a multivariable prediction model for individual prognosis or diagnosis (TRIPOD): the TRIPOD statement. BMJ. (2015) 350:g7594. doi: 10.1161/CIRCULATIONAHA.114.014508, PMID: 25569120

[25] Higgins JPT Thompson SG Deeks JJ Altman DG . Measuring inconsistency in meta-analyses. BMJ. (2003) 327:557–60. doi: 10.1136/bmj.327.7414.557, PMID: 12958120 PMC192859

[26] Egger M Davey Smith G Schneider M Minder C . Bias in meta-analysis detected by a simple, graphical test. BMJ. (1997) 315:629–34. doi: 10.1136/bmj.315.7109.629, PMID: 9310563 PMC2127453

[27] Bouganim N Dranitsaris G Hopkins S Vandermeer L Godbout L Dent S . Prospective validation of risk prediction indexes for acute and delayed chemotherapy-induced nausea and vomiting. Curr Oncol. (2012) 19:e414–421. doi: 10.3747/co.19.1074, PMID: 23300365 PMC3503672

[28] Cao Z Xiong X Yang Q . Application of naive Bayes classifier in risk prediction model of chemotherapy-induced nausea and vomiting. J South Med Univ. (2021) 41:607–12. doi: 10.12122/j.issn.1673-4254.2021.04.19, PMID: 33963723 PMC8110439

[29] Dranitsaris G Bouganim N Milano C Vandermeer L Dent S Wheatley-Price P . Prospective validation of a prediction tool for identifying patients at high risk for chemotherapy-induced nausea and vomiting. J Support Oncol. (2013) 11:14–21. doi: 10.1016/j.suponc.2012.05.001, PMID: 22763232

[30] Dranitsaris G Molassiotis A Clemons M Roeland E Schwartzberg L Dielenseger P . The development of a prediction tool to identify cancer patients at high risk for chemotherapy-induced nausea and vomiting. Ann Oncol. (2017) 28:1260–7. doi: 10.1093/annonc/mdx100, PMID: 28398530 PMC5452068

[31] Hu Z Liang W Yang Y Keefe D Ma Y Zhao Y . Personalized estimate of chemotherapy-induced nausea and vomiting: development and external validation of a nomogram in cancer patients receiving highly/moderately emetogenic chemotherapy. Medicine. (2016) 95:e2476. doi: 10.1097/MD.0000000000002476, PMID: 26765450 PMC4718276

[32] Huang G Lin H Liu Y . Analysis of risk factors of chemotherapy induced nausea and vomiting in patients with gastric cancer and construction of nomogram prediction model. Chin Gen Nurs. (2022) 20:739–43. doi: 10.12104/j.issn.1674-4748.2022.06.005

[33] Huang XJ Li XY Li JH Hu ZY Luo L Tan Y . Nomogram for predicting chemotherapy-induced nausea and vomiting for breast cancer patients. Tohoku J Exp Med. (2021) 254:111–21. doi: 10.1620/tjem.254.111, PMID: 34162779

[34] Lu X Lin C Zhang J . Establishment and evaluation of risk prediction model for nausea and vomiting induced by chemotherapy for breast cancer. Jilin Med J. (2023) 44:2807–10. doi: 10.3969/j.issn.1004-0412.2023.10.037, PMID: 35900448

[35] Molassiotis A Stamataki Z Kontopantelis E . Development and preliminary validation of a risk prediction model for chemotherapy-related nausea and vomiting. Support Care Cancer. (2013) 21:2759–67. doi: 10.1007/s00520-013-1843-2, PMID: 23715816

[36] Mosa ASM Hossain AM Yoo I . A dynamic prediction engine to prevent chemotherapy-induced nausea and vomiting. Artif Intell Med. (2020) 109:101925. doi: 10.1016/j.artmed.2020.101925, PMID: 34756214

[37] On J Park HA Yoo S . Development of a prediction models for chemotherapy-induced adverse drug reactions: A retrospective observational study using electronic health records. Eur J Oncol Nurs. (2022) 56:102066. doi: 10.1016/j.ejon.2021.102066, PMID: 34861529

[38] Zhang J Cui X Yang C Zhong D Sun Y Yue X . A deep learning-based interpretable decision tool for predicting high risk of chemotherapy-induced nausea and vomiting in cancer patients prescribed highly emetogenic chemotherapy. Cancer Med. (2023) 12:18306–16. doi: 10.1002/cam4.6428, PMID: 37609808 PMC10524079

[39] Zhang L Zeng L Sun Y Wang J Wang C Liu C . Real-world validation of the chemotherapy-induced nausea and vomiting predictive model and its optimization for identifying high-risk Chinese patients. Chin Med J (Engl). (2023) 136:1370–2. doi: 10.1097/CM9.0000000000002265, PMID: 37106525 PMC10309504

[40] Zhang Y Suo W Wu L . Analysis of influencing factors of chemotherapy-related nausea and vomiting and construction of nomogram model. Med Higher Vocational Educ Modern Nurs. (2023) 6:346–51. doi: 10.3969/j.issn.2096-501X.2023.04.017, PMID: 35900448

[41] Sheikhi MA Ebadi A Talaeizadeh A Rahmani H . Alternative methods to treat nausea and vomiting from cancer chemotherapy. Chemother Res Pract. (2015) 2015:818759. doi: 10.1155/2015/818759, PMID: 26634155 PMC4655029

[42] Clark-Snow R Affronti ML Rittenberg CN . Chemotherapy-induced nausea and vomiting (CINV) and adherence to antiemetic guidelines: results of a survey of oncology nurses. Support Care Cancer. (2018) 26:557–64. doi: 10.1007/s00520-017-3866-6, PMID: 28871358 PMC5752733

[43] Moons KG Kengne AP Grobbee DE Royston P Vergouwe Y Altman DG . Risk prediction models: II. External validation, model updating, and impact assessment. Heart. (2012) 98:691–8. doi: 10.1136/heartjnl-2011-301247, PMID: 22397946

[44] Guo H Xu K Deng F Chen Q Liang J Zhang K . Risk prediction models for preoperative deep vein thrombosis in older patients with hip fracture: A systematic review and meta-analysis. Clin Appl Thromb Hemost. (2024) 30:10760296241285565. doi: 10.1177/10760296241285565, PMID: 39318323 PMC11425752

[45] Ogundimu EO Altman DG Collins GS . Adequate sample size for developing prediction models is not simply related to events per variable. J Clin Epidemiol. (2016) 76:175–82. doi: 10.1016/j.jclinepi.2016.02.031, PMID: 26964707 PMC5045274

[46] Wynants L Bouwmeester W Moons KG Moerbeek M Timmerman D Van Huffel S . A simulation study of sample size demonstrated the importance of the number of events per variable to develop prediction models in clustered data. J Clin Epidemiol. (2015) 68:1406–14. doi: 10.1016/j.jclinepi.2015.02.002, PMID: 25817942

[47] Moons KG de Groot JA Bouwmeester W Vergouwe Y Mallett S Altman DG . Critical appraisal and data extraction for systematic reviews of prediction modelling studies: the CHARMS checklist. PloS Med. (2014) 11:e1001744. doi: 10.1371/journal.pmed.1001744, PMID: 25314315 PMC4196729

[48] Steyerberg EW Vergouwe Y . Towards better clinical prediction models: seven steps for development and an ABCD for validation. Eur Heart J. (2014) 35:1925–31. doi: 10.1093/eurheartj/ehu207, PMID: 24898551 PMC4155437

[49] Moons KG Royston P Vergouwe Y Grobbee DE Altman DG . Prognosis and prognostic research: what, why, and how? BMJ. (2009) 338:b375. doi: 10.1136/bmj.b375, PMID: 19237405

[50] Royston P Altman DG Sauerbrei W . Dichotomizing continuous predictors in multiple regression: a bad idea. Stat Med. (2006) 25:127–41. doi: 10.1002/sim.2331, PMID: 16217841

[51] Janssen KJ Donders AR Harrell FE Jr. Vergouwe Y Chen Q Grobbee DE . Missing covariate data in medical research: to impute is better than to ignore. J Clin Epidemiol. (2010) 63:721–7. doi: 10.1016/j.jclinepi.2009.12.008, PMID: 20338724

[52] Altman DG Vergouwe Y Royston P Moons KG . Prognosis and prognostic research: validating a prognostic model. BMJ. (2009) 338:b605. doi: 10.1136/bmj.b605, PMID: 19477892

[53] Bzdok D Altman N Krzywinski M . Statistics versus machine learning. Nat Methods. (2018) 15:233–4. doi: 10.1038/nmeth.4642, PMID: 30100822 PMC6082636

[54] Churpek MM Yuen TC Winslow C Meltzer DO Kattan MW Edelson DP . Multicenter comparison of machine learning methods and conventional regression for predicting clinical deterioration on the wards. Crit Care Med. (2016) 44:368–74. doi: 10.1097/CCM.0000000000001571, PMID: 26771782 PMC4736499

[55] Fu H Hou D Xu R You Q Li H Yang Q . Risk prediction models for deep venous thrombosis in patients with acute stroke: A systematic review and meta-analysis. Int J Nurs Stud. (2024) 149:104623. doi: 10.1016/j.ijnurstu.2023.104623, PMID: 37944356

[56] Yao F He L Sun X . Predictive factors of chemotherapy−induced nausea and vomiting in elderly patients with gynecological cancer undergoing paclitaxel and carboplatin therapy: A retrospective study. Oncol Lett. (2025) 29:167. doi: 10.3892/ol.2025.14913, PMID: 39958929 PMC11826289

[57] Chic N Schettini F Brasó-Maristany F Sanfeliu E Adamo B Vidal M . Oestrogen receptor activity in hormone-dependent breast cancer during chemotherapy. EBioMedicine. (2021) 69:103451. doi: 10.1016/j.ebiom.2021.103451, PMID: 34161883 PMC8233691

[58] Mosa ASM Hossain AM Lavoie BJ Yoo I . Patient-related risk factors for chemotherapy-induced nausea and vomiting: A systematic review. Front Pharmacol. (2020) 11:329. doi: 10.3389/fphar.2020.00329, PMID: 32296333 PMC7138899

[59] Di Mattei VE Carnelli L Carrara L Bernardi M Crespi G Rancoita PMV . Chemotherapy-induced nausea and vomiting in women with gynecological cancer: A preliminary single-center study investigating medical and psychosocial risk factors. Cancer Nurs. (2016) 39:E52–e59. doi: 10.1097/NCC.0000000000000342, PMID: 26895414

[60] Kawazoe H Murakami A Yamashita M Nishiyama K Kobayashi-Taguchi K Komatsu S . Patient-related risk factors for nausea and vomiting with standard antiemetics in patients with breast cancer receiving anthracycline-based chemotherapy: A retrospective observational study. Clin Ther. (2018) 40:2170–9. doi: 10.1016/j.clinthera.2018.10.004, PMID: 30392814

[61] Mukoyama N Yoshimi A Goto A Kotani H Ishikawa K Miyazaki N . An analysis of behavioral and genetic risk factors for chemotherapy-induced nausea and vomiting in Japanese subjects. Biol Pharm Bull. (2016) 39:1852–8. doi: 10.1248/bpb.b16-00440, PMID: 27803457

[62] Puri S Hyland KA Weiss KC Bell GC Gray JE Kim R . Prediction of chemotherapy-induced nausea and vomiting from patient-reported and genetic risk factors. Support Care Cancer. (2018) 26:2911–8. doi: 10.1007/s00520-018-4120-6, PMID: 29546524 PMC6200138

[63] Elguoshy A Zedan H Saito S . Machine learning-driven insights in cancer metabolomics: from subtyping to biomarker discovery and prognostic modeling. Metabolites. (2025) 15:514. doi: 10.3390/metabo15080514, PMID: 40863133 PMC12388062

[64] Tian SC Yang J Li X Huang RX Chen J . Bibliometric and visual analysis of chemotherapy-induced nausea and vomiting, (2004-2023. Front Oncol. (2024) 14:1377486. doi: 10.3389/fonc.2024.1377486, PMID: 38720800 PMC11076682

