# Supplementary material for: Risk prediction models for chemotherapy-induced nausea and vomiting: a systematic review and meta-analysis
Source: Front Oncol. 2026 Mar 10;16:1750558. doi: 10.3389/fonc.2026.1750558 (PMC13008701; doi:10.3389/fonc.2026.1750558)
Supplement: Supplementary file 1 [file DataSheet1.doc]

**Supplementary Materials**

**Appendix A. Systematic literature review search strategy in PubMed**

| Steps | Search query |
| --- | --- |
| #1 | "Neoplasms"[MESH] OR "Tumor"[All Fields] OR "Neoplasm"[All Fields] OR "Tumors"[All Fields] OR "Neoplasia"[All Fields] OR "Neoplasias"[All Fields] OR "Cancer"[All Fields] OR "Cancers"[All Fields] OR "malignant neoplasm"[All Fields] OR "Malignancy"[All Fields] OR "Malignancies"[All Fields] OR "malignant neoplasms"[All Fields] OR "neoplasm malignant"[All Fields] OR "neoplasms malignant"[All Fields] OR "benign neoplasms"[All Fields] OR "benign neoplasm"[All Fields] OR "neoplasms benign"[All Fields] OR "neoplasm benign"[All Fields] |
| #2 | "chemotherapy induced nausea vomiting"[MESH] OR "CINV"[All Fields] OR "chemotherapy nausea"[All Fields] OR "chemotherapy vomiting"[All Fields] OR "chemotherapy emesis"[All Fields] OR "nausea "[All Fields] OR "vomitting "[All Fields] OR "Retching "[All Fields] |
| #3 | "risk assessment"[MESH] OR "risk score"[All Fields] OR "risk model"[All Fields] OR "risk prediction"[All Fields] OR "scoring system"[All Fields] OR "nomogram"[All Fields] OR "prediction model"[All Fields] OR "Predictive factors"[All Fields] |
| #4 | #1AND#2AND#3 |

**Appendix B. Data collection questionnaire**

| Data collection questionnaire | |
| --- | --- |
| ***Study characteristics*** | |
| General information | Model and type of study |
|  | Secondary citations |
|  | Language of publication |
|  | Study design |
|  | Follow-up time |
| Participants | Number of included persons in the cohort |
|  | Setting |
|  | Recruitment period |
|  | Age (in years) |
|  | Sex |
|  | Stages of disease |
|  | Treatment |
|  | Inclusion criteria |
|  | Exclusion criteria |
| Predictors | List of predictors in final model (including cut-points for dichotomised factors) |
|  | Timing of predictor measurement |
| Outcome(s) | Primary outcome in study |
|  | Additional outcome(s) |
|  | Outcome in model development |
| Missing data | Participants with any missing data? |
|  | If yes, how was missing data handled? |
| Analysis | Number of participants and number of events (specific time points where reported) |
|  | Predictor selection method |
|  | Statistical method |
|  | Simplification of model? |
|  | Performance measures reported? |
|  | Creation of risk groups? |
| PROBAST:  Applicability | Domain 1: Participant selection |
|  | Domain 2: Predictors |
|  | Domain 3: Outcome |
| Notes | Funding and conflict of interest |
|  | Other comments |

| **Item** | **Authors' judgement** | **Support for judgement** |
| --- | --- | --- |
| Domain 1: Participant selection |  |  |
| Domain 2: Predictors |  |  |
| Domain 3: Outcome |  |  |
| Domain 4: Analysis |  |  |
| Overall judgement |  |  |

**Appendix C. PROBAST questionnaire**

| PROBAST questionnaire |  |
| --- | --- |
| **DOMAIN 1: Participants**  *A. Risk of Bias*  Describe the sources of data and criteria for participant selection:  1.1 Were appropriate data sources used, e.g. cohort, RCT or nested case-control study data?  1.2 Were all inclusions and exclusions of participants appropriate?  Risk of bias introduced by selection of participants RISK: (low/ high/ unclear)  Rationale of bias rating:  *B. Applicability*  Describe included participants, setting and dates:  Concern that the included participants and setting do not match the review question:  CONCERN: (low/ high/ unclear)  Rationale of applicability rating: | |
| **DOMAIN 2: Predictors**  *A. Risk of Bias*  List and describe predictors included in the final model, e.g. definition and timing of assessment:  2.1 Were predictors defined and assessed in a similar way for all participants?  2.2 Were predictor assessments made without knowledge of outcome data?  2.3 Are all predictors available at the time the model is intended to be used?  Risk of bias introduced by predictors or their assessment RISK: (low/ high/ unclear)  Rationale of bias rating:  *B. Applicability*  Concern that the definition, assessment or timing of predictors in the model do not match the review  question:  CONCERN: (low/ high/ unclear)  Rationale of applicability rating: | |
| **DOMAIN 3: Outcome**  *A. Risk of Bias*  Describe the outcome, how it was defined and determined, and the time interval between  predictor assessment and outcome determination:  3.1 Was the outcome determined appropriately?  3.2 Was a pre-specified or standard outcome definition used?  3.3 Were predictors excluded from the outcome definition?  3.4 Was the outcome defined and determined in a similar way for all participants?  3.5 Was the outcome determined without knowledge of predictor information?  3.6 Was the time interval between predictor assessment and outcome determination appropriate?  Risk of bias introduced by the outcome or its determination RISK: (low/ high/ unclear)  Rationale of bias rating:  *B. Applicability*  At what time point was the outcome determined:  If a composite outcome was used, describe the relative frequency/distribution of each contributing  outcome:  Concern that the outcome, its definition, timing or determination do not match the review  question:  CONCERN: (low/ high/ unclear)  Rationale of applicability rating: | |
| **DOMAIN 4: Analysis**  *Risk of Bias*  Describe numbers of participants, number of candidate predictors, outcome events and events per  candidate predictor:  Describe how the model was developed (for example in regards to modelling technique (e.g. survival  or logistic modelling), predictor selection, and risk group definition):  Describe whether and how the model was validated, either internally (e.g. bootstrapping, cross  validation, random split sample) or externally (e.g. temporal validation, geographical validation,  different setting, different type of participants):  Describe the performance measures of the model, e.g. (re)calibration, discrimination,  (re)classification, net benefit, and whether they were adjusted for optimism:  Describe any participants who were excluded from the analysis:  Describe missing data on predictors and outcomes as well as methods used for missing data:  4.1 Were there a reasonable number of participants with the outcome?  4.2 Were continuous and categorical predictors handled appropriately?  4.3 Were all enrolled participants included in the analysis?  4.4 Were participants with missing data handled appropriately?  4.5 Was selection of predictors based on univariable analysis avoided?  4.6 Were complexities in the data (e.g. censoring, competing risks, sampling of controls) accounted  for appropriately?  4.7 Were relevant model performance measures evaluated appropriately?  4.8 Were model overfitting and optimism in model performance accounted for?  4.9 Do predictors and their assigned weights in the final model correspond to the results from  multivariable analysis?  Risk of bias introduced by the analysis RISK: (low/ high/ unclear)  Rationale of bias rating: | |

**Appendix D. TRIPOD checklist**

| **Section/Topic** | **Item** | **Development or Validation?** | **Checklist Item** |
| --- | --- | --- | --- |
| **Title and abstract** |  |  |  |
| Title | 1 | D;V | Identify the study as developing and/or validating a multivariable prediction model, the target population, and the outcome to be predicted. |
| Abstract | 2 | D;V | Provide a summary of objectives, study design, setting, participants, sample size, predictors, outcome, statistical analysis, results, and conclusions. |
| **Introduction** |  |  |  |
| Background and  objectives | 3a | D;V | Explain the medical context (including whether diagnostic or prognostic) and rationale for  developing or validating the multivariable prediction model, including references to existing models. |
|  | 3b | D;V | Specify the objectives, including whether the study describes the development or validation of the model, or both. |
| **Methods** |  |  |  |
| Source of data | 4a | D;V | Describe the study design or source of data (e.g., randomized trial, cohort, or registry data), separately for the development and validation datasets, if applicable. |
|  | 4b | D;V | Specify the key study dates, including start of accrual; end of accrual; and, if applicable, end of follow-up. |
| Participants | 5a | D;V | Specify key elements of the study setting (e.g., primary care, secondary care, general population) including number and location of centres. |
|  | 5b | D;V | Describe eligibility criteria for participants. |
|  | 5c | D;V | Give details of treatments received, if relevant. |
| Outcome | 6a | D;V | Clearly define the outcome that is predicted by the prediction model, including how and when assessed. |
|  | 6b | D;V | Report any actions to blind assessment of the outcome to be predicted. |
| Predictors | 7a | D;V | Clearly define all predictors used in developing the multivariable prediction model, including how and when they were measured. |
|  | 7b | D;V | Report any actions to blind assessment of predictors for the outcome and other predictors. |
| Sample size | 8 | D;V | Explain how the study size was arrived at. |
| Missing data | 9 | D;V | Describe how missing data were handled (e.g., complete-case analysis, single imputation, multiple imputation) with details of any imputation method. |
| Statistical analysis methods | 10a | D | Describe how predictors were handled in the analyses. |
|  | 10b | D | Specify type of model, all model-building procedures (including any predictorselection), and method for internal validation. |
|  | 10c | V | For validation, describe how the predictions were calculated. |
|  | 10d | D;V | Specify all measures used to assess model performance and, if relevant, to compare multiple models. |
|  | 10e | V | Describe any model updating (e.g., recalibration) arising from the validation, if done. |
| Risk groups | 11 | D;V | Provide details on how risk groups were created, if done. |
| Development vs.  validation | 12 | V | For validation, identify any differences from the development data in setting, eligibility criteria, outcome, and predictors. |
| Results |  |  |  |
| Participants | 13a | D;V | Describe the flow of participants through the study, including the number of participants with and without the outcome and, if applicable, a summary of the follow-up time. A diagram maybe  helpful. |
|  | 13b | D;V | Describe the characteristics of the participants (basic demographics, clinical features, available predictors), including the number of participants with missing data for predictors and outcome. |
|  | 13c | V | For validation, show a comparison with the development data of the distribution of important variables (demographics, predictors, and outcome). |
| Model development | 14a | D | Specify the number of participants and outcome events in each analysis. |
|  | 14b | D | If done, report the unadjusted association between each candidate predictor and outcome. |
| Model specification | 15a | D | Present the full prediction model to allow predictions for individuals (i.e., all regression coefficients, and model interceptor baseline survival at a given time point). |
|  | 15b | D | Explain how to use the prediction model. |
| Model performance | 16 | D;V | Report performance measures (with CIs) for the prediction model. |
| Model updating | 17 | V | If done, report the results from any model updating (i.e., model specification, model performance). |
| **Discussion** |  |  |  |
| Limitations | 18 | D;V | Discuss any limitations of the study (such as nonrepresentative sample, few events per predictor, missing data). |
| Interpretations | 19a | V | For validation, discuss the results with reference to performance in the development data, and any other validation data. |
|  | 19b | D;V | Give an overall interpretation of the results, considering objectives, limitations, results from similar studies, and other relevant evidence. |
| Implications | 20 | D;V | Discuss the potential clinical use of the model and implications for future research. |
| **Other information** |  |  |  |
| Supplementary  information | 21 | D;V | Provide information about the availability of supplementary resources, such as study protocol, Web calculator, and datasets. |
| Funding | 22 | D;V | Give the source of funding and the role of the funders for the present study. |

**Appendix E. Overview of the information of the included prediction models**

| **Overview of the information of the included prediction models** | | | | | | | | | | | | | |
| --- | --- | --- | --- | --- | --- | --- | --- | --- | --- | --- | --- | --- | --- |
| Author  (Year) | Model development method | Variable selection | Candidate predictors | | Missing data | | Validation method | Model performance | Calibration method | Final predictors | Model presentation | Strengths | Limitations |
|  |  |  | Number | Continuous variable processing method | Number | Missing data handling |  |  |  |  |  |  |  |
| Lu xiangyuan*  (2023) | multivariable logistic regression model | Stepwise Regression Analysis | 17 | Categorical variables | - | - | Internal validation  (bootstrap sampling) | A:0.72(0.672-0.783) | Hosmer-Lemeshow test | age,  previous history of CINV,  anxiety state,  numbers of chemotherapy | Nomogram | The visualization of results can accurately and intuitively predict the probability of an individual occurrence of a certain clinical event or outcome, which is conducive to the screening of high-risk groups and the formulation of clinical decisions. | Single-center study lacking external validation. |
| Huang guiling*  (2022) | multivariable logistic regression model | Stepwise Regression Analysis | 18 | Categorical variables | - | - | Internal validation  (bootstrap sampling) | A:0.759(0.688-0.830)  B:0.776(0.704-0.849) | Hosmer-Lemeshow test | age,  gender,  history of alcohol consumption,  previous history of CINV,  numbers of chemotherapy | Nomogram | The nomogram has the advantages of intuitive, concise, visual and convenient, which is conducive to providing clinicians with a simple and fast prediction tool. | Single-center study with relatively few cases; no external validation was performed. |
| Zhang yuqing*  (2023) | multivariable logistic regression model | Stepwise Regression Analysis | 19 | Categorical variables | 12 | Direct exclusion | - | - | - | types of antiemetic drugs,  age,  gender,  previous history of CINV,  numbers of chemotherapy, pregnancy-related nausea and vomiting,  sleep quality score,  anticipatory nausea and vomiting, | Nomogram model | The standard is relatively simple, which is conducive to the use of grassroots hospitals. | The sample size was small and failed to be used.  The constructed nomogram model was externally validated with independent samples. |
| Deng benmin*  (2022) | multivariable logistic regression model | Stepwise Regression Analysis | 31 | Categorical variables | 92 | Direct exclusion | - | A:0.843(0.825-0.861) | Hosmer-Lemeshow test | platinum-based chemotherapy regimen,  AC-containing chemotherapy regimen,  sleep time was < 7 h the night before chemotherapy,  CINV prodromal symptoms,  CVASS-A Score≥ 3,  pain,  age< 58,  history of alcohol consumption,  VAS ≥30 mm,  previous history of CINV,  anticipatory nausea and vomiting | Formula of risk score obtained by partial regression coefficient of each factor | Covers chemotherapy drugs and patients  The individual risk factors have a good degree of differentiation and accuracy. | Lack of external validation. |
| Cao zongping*  (2021) | Naive Bayesian classifier | - | 26 | Categorical variables | - | - | Cross-validation  & Random split validation | Acute-auc:0.72±0.04(0.69-0.75);  Delayed-auc:0.74 ± 0.02  (0.72-0.77) | - | age,  whether there has been a pregnancy vomiting reaction,  history of alcohol consumption,  presence of underlying disease,  tumor type,  tumor stage,  numbers of chemotherapy,  patients who are not the first chemotherapy patient,  previous history of CINV,  anticipatory nausea and vomiting,  anxiety level,  whether the chemotherapy regimen uses highly emetic drugs,  sleep duration at night before chemotherapy,  whether or not glucocorticoids before chemotherapy,  whether 5-HT3 is used before chemotherapy,  whether NK-1 was used prior to chemotherapy,  whether to use glucocorticoids after chemotherapy, whether 5-HT3 is used after chemotherapy | - | The naive Bayesian classifier was applied to Chinese patients for the first time  In the risk prediction of CINV, the model incorporates individual risk factors and takes them into account  treatment protocol to identify high-risk patients. | Model optimization, external validation, and comparative experiments with larger sample sizes are required. |
| Hu zhihuang  (2016) | multivariable logistic regression model | Stepwise Regression Analysis | 9 | Categorical variables | 19 | Direct exclusion | Internal validation  (bootstrap sampling) & External Verification (Spatial Verification) | C:0.67(0.62–0.72)  D:0.65(0.58–0.72) | Calibration curves | gender,  age,  history of alcohol consumption,  history of vomiting pregnancy,  previous history of CINV,  body surface area, emetogenicity of chemotherapy,  antiemetic regimens | Nomogram | User-friendly interface and visual graphics, easy to use, and external verification in different countries. | All patients in the study were from Asian countries, so its applicability in other ethnic populations should be further investigated. |
| Bouganim (2012) | - | - | - | - | - | - | External Verification (Spatial Verification) | Acute-auc:0.69;(0.59-0.79)  Delayed-auc:0.70(0.60-0.80) | Hosmer-  Lemeshow test | Acute:  age > 40,  presence of complications,  history of alcohol consumption,  3rd or more cycle chemotherapy,  gynecologic tumors or gastrointestinal tumors,  anthracycline-based chemotherapy regimens,  platinum-based chemotherapy regimens,  stage I or II tumors,  over-the-counter antiemetics used at home  Delayed:  use of class 5-HT3 antiemetic medication with or without dexamethasone,  previous history of CINV,  pregnancy reactions during pregnancy,  use of over-the-counter antiemetic therapy at home,  more than 1 vomiting in the acute phase,  3rd or more cycles of chemotherapy,  sleep duration the night before chemotherapy | - | The risk index performed relatively well. They are easy to apply and are able to distinguish between high-risk and low-risk patients. | The sample size was small, and patient data were obtained from a single institution. |
| Mosa  (2020) | machine-learning algorithms  (decision tree) | - | 26 | Categorical variables | - | - | Cross-validation | - | - | age,  gender,  race,  BMI,  type of cancer,  stage of cancer,  number of comorbidities,  history of alcohol consumption,  current smoker,  anxiety,  dehydration,  chemotherapy regimens,  previous history of CINV | CINV decision support system | a highly accurate prediction system that is dynamic and updateable in real-time. | Lack of external validation. |
| Dranitsaris G  (2017) | GEE model | Backwards elimination | 16 | Categorical variables | - | - | Internal validation  (bootstrap sampling) | A:0.69(0.67-0.70) | Hosmer-  Lemeshow test | age <60,  the first two cycles of chemotherapy,  anticipatory nausea and vomiting,  history of morning sickness,  hours of sleep the night before chemotherapy,  previous history of CINV,  self-medication with non-prescribed treatments,  the use of platinum or anthracycline-based regimens | Risk score rating | Cycle-based repeated measures model. | Lack of external validation and low specificity (38.4%) |
| Molassiotis (2013) | Multivariable logistic regression model | Stepwise Regression Analysis | 16 | Continuous variable | - | multiple imputation  approach | Internal validation  (bootstrap sampling & Random split validation) | - | - | age,  previous history of CINV,  anxiety,  symptom distress, antiemetic type | A dynamic web-based  tool | For the first time, patients with prodromal symptoms of chemotherapy were included in the risk assessment. | The sample size is small and only outpatients are used, and external validation is lacking. |
| Huang Xinjuan (2021) | Multivariable logistic regression model | Stepwise Regression Analysis | 25 | Categorical variables | 20 | Direct exclusion | Internal validation  (bootstrap sampling) &  External Verification (Spatial Verification) | C:0.78(0.73-0.83)  D:0.71(0.58-0.84) | Calibration curves | previous history of CINV,  chemotherapy regimen, chemotherapy cycle, metastasis,  symptoms of distress | Nomogram | This tool has a graphical interface and is thus easy to use; individual patients’ CINV risk can be determined without complex mathematical calculations. | The sample size was small and only included Chinese participants. |
| Zhang Linlin (2023) | GEE model | Stepwise Regression Analysis | 10 | Categorical variables | - | - | External Verification (Spatial Verification) | C:0.629(0.592-0.665)  D:0.685(0.631-0.738) | Hosmer-  Lemeshow test | age <60,  anticipatory nausea and vomiting,  history of morning sickness,  use of antiemetics at home,  previous history of CINV, | Risk scoring system | this study performed real-world validation and optimization of a highly-recommended CINV predictive model in Chinese patients. | The optimized predictive model excludes the anticancer agent itself. |
| Dranitsaris G  (2013) | - | - | - | Categorical variables | 78 | Direct exclusion | External Verification (Spatial Verification) | Acute-auc:0.70(0.62-0.77)  Delayed-auc:0.75(0.69-0.80) | Hosmer-Lemeshow test | Acute:  age > 40,  presence of complications,  history of alcohol consumption,  3rd or more cycle chemotherapy,  gynecologic tumors or gastrointestinal tumors,  anthracycline-based chemotherapy regimens,  platinum-based chemotherapy regimens,  stage I or II tumors,  over-the-counter antiemetics used at home  Delayed:  use of class 5-HT3 antiemetic medication with or without dexamethasone,  previous history of CINV,  pregnancy reactions during pregnancy,  use of over-the-counter antiemetic therapy at home,  more than 1 vomiting in the acute phase,  3rd or more cycles of chemotherapy,  sleep duration the night before chemotherapy |  | The indexes are easy to  apply and can discriminate between high- and low-risk  patients, and the threshold can be varied depending on a  patient’s and/or clinician’s risk tolerance. | The sample size was small, also considered only cancer patients  receiving outpatient chemotherapy. As a result, the indexes may not be applicable to hospitalized patients. |
| Jeongah On  (2021) | machine-learning algorithms  (logistic regression, decision tree, and artificial neural network) | Univariate analyses | 63 | Categorical variables | - | - | Internal validation  (Cross-validation) | A:0.83 | - | age,  gender,  cancer type,  chemotherapy type,  ADRs history,  comorbidities | Automatic calculation of risk scoring system | More predictors that have not been used in previous studies are included. | Lack of clinical validation of validity. |
| Zhang jingyue  (2023) | Deep learning algorithms | SMOTE | 20 | SMOTE oversampling | 10 | MICE method | Internal validation | A:0.850(0.780-0.919) | Calibration curves, Brier scores | Ccr,  age,  gender,  anticipatory nausea and vomiting,  antiemetic regimen | Automatic calculation of risk scoring system | This model outperforms typical machine learning models and incorporates liver and kidney function in conjunction with known individual variables | The lack of external validation and the study population consisting solely of Chinese patients. |
| "-"= Not reported; A=AUC; B=C-index; C=Development cohort; D=Validation cohort.  BMI=body mass index, Ccr=creatinine clearance, SMOTE=Synthetic Minority Oversampling Technique, MICE=Multivariate Imputation Chained Equations, GGE=generalized estimating equation.  AUC= Area under the curve. We considered AUC = 0.5–0.7 as poor discrimination, 0.7–0.8 as moderate discrimination, 0.8–0.9 as good discrimination, and 0.9–1.0 as excellent discrimination. | | | | | | | | | | | | | |

**Appendix F. Included studies were assessed according to the TRIPOD**

| **Included studies were assessed according to the TRIPOD** | | | | | | | | | | | | | | |
| --- | --- | --- | --- | --- | --- | --- | --- | --- | --- | --- | --- | --- | --- | --- |
| Author (Year) | Title | Abstract | Introduction | Objectives | Study dsign | Study date | Study setting | eligibility criteria | treatment,if relevant | outcome definition | blind assessment of outcome | Definition of predictors | blind assessment of predictors | study size |
|  | 1 | 2 | 3a | 3b | 4a | 4b | 5a | 5b | 5c | 6a | 6b | 7a | 7b | 8 |
| Lu xiangyuan*(2023) | Y | N | N | Y | Y | Y | Y | Y | Y | Y | N | Y | Y | N |
| Huang guiling* (2022) | Y | Y | N | Y | Y | Y | Y | Y | Y | Y | N | Y | Y | N |
| Zhang yuqing*(2023) | N | Y | N | Y | Y | N | Y | Y | N | Y | N | Y | Y | N |
| Deng benmin*(2022) | N | Y | Y | Y | Y | Y | Y | Y | Y | Y | N | Y | Y | Y |
| Cao zongping*(2021) | N | Y | Y | Y | Y | Y | Y | Y | Y | Y | N | Y | N | N |
| Hu zhihuang(2016) | Y | Y | Y | Y | Y | Y | Y | Y | Y | Y | N | Y | Y | N |
| Bouganim (2012) | N | Y | N | Y | Y | Y | Y | Y | Y | Y | N | Y | Y | N |
| Mosa(2021) | Y | Y | N | Y | Y | Y | Y | Y | Y | Y | N | Y | N | N |
| Dranitsaris G(2017) | Y | Y | Y | Y | Y | Y | N | Y | N | Y | N | Y | N | N |
| Molassiotis (2013) | N | N | Y | Y | Y | Y | Y | Y | Y | Y | N | Y | Y | Y |
| Huang Xinjuan (2021) | Y | Y | Y | Y | Y | Y | Y | Y | Y | Y | N | Y | Y | N |
| Zhang Linlin (2023) | N | N | Y | Y | Y | Y | Y | N | N | Y | N | Y | Y | N |
| Dranitsaris G(2013) | Y | N | N | Y | Y | Y | Y | Y | N | Y | N | Y | Y | N |
| Jeongah On(2021) | N | Y | N | Y | Y | N | Y | Y | Y | Y | N | Y | N | N |
| Zhang jingyue(2023) | Y | Y | N | Y | Y | Y | Y | Y | N | Y | N | Y | N | N |
| Y=yes , N=no, NA=not applicable | | | | | | | | | | | | | | |

| **Continuation table-Included studies were assessed according to the TRIPOD** | | | | | | | | | | | | | |
| --- | --- | --- | --- | --- | --- | --- | --- | --- | --- | --- | --- | --- | --- |
| Author (Year) | Handling missing data | Handling predictors | model development procedure | Predictor calculation | assessing model performance | model updating | risk groups | any differences from model development study | Results: flow of participants selection | Participant characteristics | Comparison with model development data | number of outcome | unadjusted association, if done |
|  | 9 | 10a | 10b | 10c | 10d | 10e | 11 | 12 | 13a | 13b | 13c | 14a | 14b |
| Lu xiangyuan*(2023) | N | N | Y | NA | Y | NA | NA | NA | Y | Y | NA | Y | Y |
| Huang guiling* (2022) | N | N | Y | NA | Y | NA | NA | N | Y | Y | NA | Y | Y |
| Zhang yuqing*(2023) | N | N | Y | NA | N | NA | NA | N | Y | Y | NA | Y | Y |
| Deng benmin*(2022) | Y | Y | Y | NA | Y | NA | Y | N | Y | Y | NA | Y | Y |
| Cao zongping*(2021) | N | Y | Y | NA | Y | NA | NA | Y | Y | Y | NA | N | N |
| Hu zhihuang(2016) | Y | Y | Y | Y | Y | Y | NA | Y | Y | Y | Y | Y | Y |
| Bouganim (2012) | N | NA | NA | Y | Y | Y | Y | N | Y | Y | N | NA | NA |
| Mosa(2021) | Y | Y | Y | NA | N | NA | Y | N | Y | Y | NA | Y | N |
| Dranitsaris G(2017) | N | N | Y | NA | Y | NA | NA | N | Y | Y | NA | Y | Y |
| Molassiotis (2013) | Y | Y | Y | Y | N | Y | NA | Y | Y | Y | Y | Y | Y |
| Huang Xinjuan (2021) | N | N | Y | Y | Y | Y | NA | Y | Y | Y | Y | Y | Y |
| Zhang Linlin (2023) | N | N | Y | Y | Y | Y | NA | N | N | N | N | N | Y |
| Dranitsaris G(2013) | N | NA | NA | Y | Y | Y | Y | N | Y | Y | Y | NA | NA |
| Jeongah On(2021) | N | Y | Y | NA | Y | NA | NA | NA | Y | Y | NA | Y | Y |
| Y=yes , N=no, NA=not applicable | | | | | | | | | | | | | |

| **Continuation table-Included studies were assessed according to the TRIPOD** | | | | | | | | | | |
| --- | --- | --- | --- | --- | --- | --- | --- | --- | --- | --- |
| Author (Year) | full model presention | How to use the model | Performance measures | Model updating, if done | Discussion: study limitations | Comparison with the model development study | Overall interpretation | Potential clinical use | Supplementary information | Funding |
|  | 15a | 15b | 16 | 17 | 18 | 19a | 19b | 20 | 21 | 22 |
| Lu xiangyuan*(2023) | Y | Y | Y | NA | N | NA | Y | Y | N | N |
| Huang guiling* (2022) | Y | Y | Y | NA | Y | NA | Y | Y | N | N |
| Zhang yuqing*(2023) | Y | Y | N | NA | Y | NA | Y | Y | N | N |
| Deng benmin*(2022) | Y | N | Y | NA | Y | NA | Y | Y | N | Y |
| Cao zongping*(2021) | N | N | Y | NA | Y | NA | Y | Y | N | Y |
| Hu zhihuang(2016) | Y | Y | Y | NA | Y | N | Y | Y | N | Y |
| Bouganim (2012) | NA | NA | Y | NA | Y | Y | N | Y | N | Y |
| Mosa(2021) | Y | N | Y | NA | Y | NA | Y | Y | N | Y |
| Dranitsaris G(2017) | Y | Y | Y | NA | Y | NA | Y | Y | N | Y |
| Molassiotis (2013) | Y | Y | N | NA | Y | N | Y | Y | N | N |
| Huang Xinjuan (2021) | Y | Y | Y | NA | Y | Y | Y | Y | N | Y |
| Zhang Linlin (2023) | Y | Y | Y | Y | Y | Y | Y | Y | Y | Y |
| Dranitsaris G(2013) | NA | NA | Y | NA | Y | Y | Y | Y | N | Y |
| Jeongah On(2021) | Y | N | N | NA | Y | NA | Y | Y | Y | Y |
| Y=yes , N=no, NA=not applicable | | | | | | | | | | |

**Appendix G. Result of Begg's test and Egger's test**


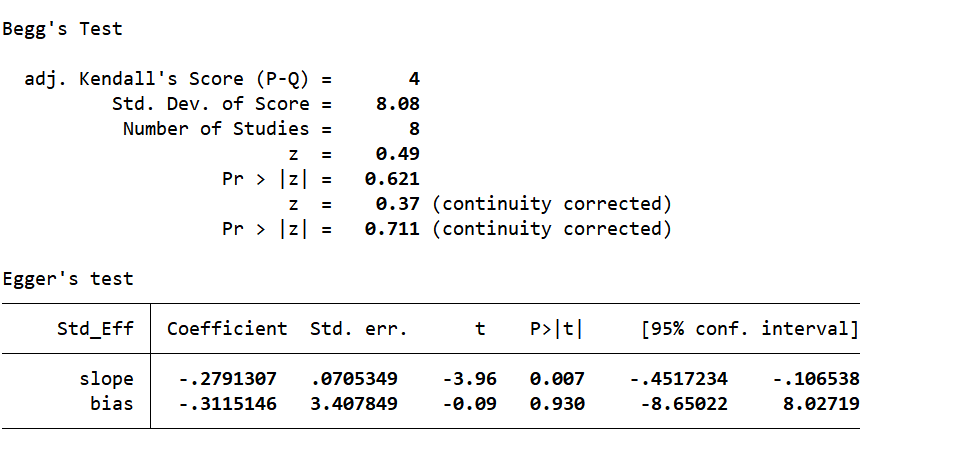


**Appendix H. Subgroup analysis**

| Subgroup analysis of the AUC values of the models | | | | | | | | | |
| --- | --- | --- | --- | --- | --- | --- | --- | --- | --- |
| Subgroups | Number of studies | Effect model | Pooled mean score |  | Effect size |  | Heterogeneity |  | Test for  subgroup  difference |
|  |  |  | (95%CI) |  | Z (p) |  | I^2^ (p) |  | Z df  (p) |
| **Chemotherapy regimen** |  |  |  |  |  |  |  |  |  |
| MEC | 2 | Fixed | 0.74(0.69,0.78) |  | 33.32(<0.001) |  | 0.0 % (0.379) |  | Z=24.25 df=7  (*P*<0.001) |
| HEC | 2 | Fixed | 0.84(0.82,0.86) |  | 85.69(<0.001) |  | 0.0 % (0.788) |  |
| MEC and HEC | 1 | - | - |  | 26.26 (<0.001) |  | - |  |
| LEC, MEC and HEC | 3 | Random | 0.72(0.66,0.77) |  | 25.68 (<0.001) |  | 82.6% (0.003) |  |
| **Cancer type** |  |  |  |  |  |  |  |  |  |
| Breast cancer | 2 | Fixed | 0.75(0.72,0.79) |  | 25.06 (<0.001) |  | 49.0 % (0.114) |  | Z=24.25 df=7  (*P*<0.001) |
| Mixed cancer types | 5 | Random | 0.75(0.66,0.83) |  | 17.20 (<0.001) |  | 97.5% (0.000) |  |
| Gastric cancer | 1 | - | - |  | 21.28 (<0.001) |  | - |  |
| **Model development method** |  |  |  |  |  |  |  |  |  |
| multivariable logistic regression model | 5 | Random | 0.76(0.68,0.83) |  | 20.82 (<0.001) |  | 92.2% (0.000) |  | Z=24.25 df=7  (*P*<0.001) |
| GEE model | 2 | Fixed | 0.69(0.68,0.70) |  | 93.45 (<0.001) |  | 0.0 % (1.000) |  |
| Deep learning algorithms | 1 | - | - |  | 23.80 (<0.001) |  | - |  |
| **CINV definition and assessment tool** |  |  |  |  |  |  |  |  |  |
| MAT | 5 | Random | 0.74(0.67,0.82) |  | 18.35 (<0.001) |  | 97.2% (0.000) |  | Z=24.25 df=7  (*P*<0.001) |
| Other | 3 | Random | 0.76(0.65,0.86) |  | 13.90 (<0.001) |  | 88.5% (0.000) |  |
| Note: -: Not report; HEC: highly emetogenic chemotherapy; MEC: moderately emetogenic chemotherapy; LEC: lowly emetogenic chemotherapy; GEE=generalized estimating equation; MAT: MASCC antiemesis tool. | | | | | | | | | |
